# Robotic versus laparoscopic anterior resection of sigmoid colon cancer: comparative study of long-term oncologic outcomes

**DOI:** 10.1007/s00464-012-2619-3

**Published:** 2012-12-13

**Authors:** Dae Ro Lim, Byung Soh Min, Min Sung Kim, Sami Alasari, Gangmi Kim, Hyuk Hur, Seung Hyuk Baik, Kang Young Lee, Nam Kyu Kim

**Affiliations:** Section of Colon and Rectal Surgery, Department of Surgery, Yonsei University College of Medicine, Seoul, South Korea

**Keywords:** Minimally invasive surgery, Robotic surgery, Sigmoid colon cancer

## Abstract

**Background:**

Robotically assisted colon resection is a new type of surgery for colon cancer. However, the evidence is inadequate for the general adaptation of robotic colon surgery. This study aimed to show the oncologic and perioperative clinical results of robotically assisted anterior resection (R-AR) compared with those of laparoscopically assisted anterior resection (L-AR) for sigmoid colon cancer.

**Methods:**

A total of 180 patients (sigmoid colon cancer stages 1–3) were assigned to receive either R-AR (*n* = 34) or L-AR (*n* = 146) between April 2006 and September 2008. Patient characteristics, perioperative clinical results, and long-term oncologic outcomes were compared between the two groups.

**Results:**

The patient characteristics did not differ significantly between the two groups. The mean operation time was 217.6 ± 70.7 min for L-AR versus 252.5 ± 94.9 min for R-AR (*p* = 0.016). The total postoperative complication rate was 10.3 % for R-AR versus 5.9 % for L-AR (*p* = 0.281). The 3-year overall survival rate for all the patients was 93.4 % for L-AR versus 92.1 % for R-AR (*p* = 0.723). The 3-year overall survival rate was 100 % for both L-AR and R-AR in stage 1, 95.5 % for L-AR versus 100 % for R-AR (*p* = 0.386) in stage 2, and 88.4 % for L-AR versus 72.9 % (*p* = 0.881) for R-AR in stage 3.

**Conclusion:**

In this study, R-AR showed safety and feasibility in terms of perioperative clinical and long-term oncologic outcomes. However, the advanced technologies of R-AR did not translate into better long-term oncologic outcomes compared with L-AR.

Laparoscopically assisted colectomy has been accepted as a safe minimally invasive procedure since several large-scale randomized clinical trials reported the oncologic safety and better short-term outcomes compared with those of open surgery [1–3].

Robotically assisted colectomy, introduced by Weber and coworker [4] in 2001, can be understood as a new variation of laparoscopic surgery because the robotic system uses video laparoscopy and pneumoperitoneum, which are similar in nature to conventional laparoscopic surgery. However, the robotic system has several advanced technologies compared with conventional laparoscopic surgery.

The technological advantages of the robotic system are a three-dimensional surgical view using a stable camera platform, fine and free movements of the robotic arm in the surgical fields, tremor elimination, motion scaling, dexterity, and ambidextrous capability [5, 6]. It has been interesting to observe how these advanced robotic technologies affect the short- and long-term outcomes of colon cancer surgery compared with conventional laparoscopic surgery.

Reports from several case series have described the feasibility and safety of robotic surgery for colon cancer [5–9]. However, to date, comparative results in terms of long-term oncologic outcomes after robotic surgery for colon cancer are scarce. This study aimed to evaluate the long-term oncologic outcomes and perioperative short-term clinical outcomes compared with the results of conventional laparoscopic surgery for colon cancer.

## Materials and methods

Between April 2006 and December 2008, 334 sigmoid colon cancer patients (stages 1–3 adenocarcinoma) underwent a curative anterior resection at Severance Hospital, Yonsei University, Seoul, South Korea. Of these 334 patients, 180 consecutive patients with a diagnosis of sigmoid colon cancer underwent curative minimally invasive surgery, which included laparoscopically assisted anterior resection (L-AR, *n* = 146) and robotically assisted anterior resection (R-AR, *n* = 34) using the da Vinci surgical system (Intuitive Surgical, Sunnyvale, CA, USA), and 154 patients underwent open surgery according to the decision of the patient with his or her informed consent. This study enrolled 180 patients who underwent either L-AR or R-AR.

The data were collected prospectively from the Yonsei Colorectal Cancer Database. The perioperative clinical results and oncologic outcomes were compared retrospectively between the L-AR group and the R-AR group. The oncologic data were updated again for the current study using electronic medical charts and telephone interviews.

Patient characteristics, American Society of Anesthesiologists (ASA) score [10], history of previous abdominal surgeries, and body mass index (BMI) were evaluated. The criteria for discharge specified no apparent complications, no abnormal physical examination findings, no subjective complaints, and tolerance of a soft diet.

Postoperative complications were categorized using the Accordion severity-grading system [11]. Conversion was defined as the need for a laparotomy exceeding the routine length of an incision for specimen extraction (4 cm) at any time to complete the entire surgical procedure.

For the postoperative pathologic results, tumor node metastasis (TNM) stage (American Joint Committee on Cancer [AJCC] 6th) [12], grade of tumor differentiation, distal and proximal resection margins, and number of harvested lymph nodes were evaluated. Recurrence was defined as the presence of radiologically confirmed or histologically proven tumor. Follow-up assessments of the patients were performed routinely at 1, 3 months, and then every 3 months until 3 years and every 6 months until 5 years. Chest and abdominopelvic computed tomography (CT) scan were used for local detection or systemic recurrence every 6 months.

This study was approved by the Institutional Review Board of Severance Hospital, and informed consent was obtained from all the patients.

### Surgical technique of laparoscopically assisted anterior resection

A standard mechanical bowel preparation was performed 24 h before the operation. The patient was placed in a modified lithotomy position with the legs apart. After achievement of pneumoperitoneum (pressure, 12 mmHg), a 12-mm trocar was placed through an incision just above the umbilicus. A 30° laparoscope then was inserted through the 12-mm trocar. The second 12-mm trocar was inserted at the lower right quadrant of the abdomen. The third 5-mm trocar was inserted at the upper right quadrant of the abdomen. The fourth and fifth 5-mm trocars were inserted at the upper and the lower left quadrants. The patient then was placed in a modified lithotomy position with the legs apart in a 30° Trendelenburg position with the right side down at 15^o^.

After ligation of the inferior mesenteric artery and vein, medial-to-lateral dissection is performed to the left lateral gutter. The sigmoid colon itself was divided using an endo-GIA (Covidien Echelon). The specimen was extracted though the left lower trocar incision, which was enlarged to ~3–4 cm, after protection. An end-to-end anastomosis (EEA) anvil then was inserted into the proximal colon and secured with a purse-string suture. The colon was placed back into the abdomen, and the port site was closed. Pneumoperitoneum was restored, and a circular stapler was used to create an end-to-end anastomosis.

### Surgical technique of robotically assisted anterior resection

After induction of general anesthesia, the patient was placed in a modified lithotomy position with the legs apart. A 12-mm trocar was placed through an incision just below the umbilicus after achievement of pneumoperitoneum. A 30° standard 12-mm robotic laparoscope then was inserted through the 12-mm trocar. The first 8-mm da Vinci trocar was placed in the middle point on the line between the infraumbilical 12-mm trocar and the right anterior superior iliac supine. The second 8-mm da Vinci trocar was inserted into the right upper abdomen. The third 8-mm da Vinci trocar was inserted 2 cm below the xiphoid process. The 11-mm trocar was placed in the right midabdomen lateral to the umbilicus and 3 cm lateral from the midaxillary line to allow access of the assistant for mobilization of the left colon. The remainder of the procedure was the same as in laparoscopic surgery except for the specimen extraction site and the infraumblilical trocar, which was enlarged to 3–4 cm. An EEA was used in both the laparoscopic and robotic procedures.

### Statistical analysis

Data were analyzed using the SPSS statistical program (Statistical Product and Service Solution 18 for Windows; SPSS Inc., Chicago, IL, USA). The Chi-square test for categorical variables and the Student’s *t* test or the Mann–Whitney test for continuous variables were used for statistical comparisons of perioperative clinical outcomes. Cumulative-incidence methods were used to estimate the rate of cancer recurrence. Overall survival and disease-free survival were analyzed using the Kaplan–Meier method, and a comparison was performed using the log-rank test. Converted cases were considered on an intention-to-treat basis. All *p* values lower than 0.05 were considered statistically significant.

## Results

### Patient characteristics

In this study, 146 patients underwent L-AR, and 34 patients underwent R-AR. The following parameters were evaluated as the characteristics of the patients: age, sex, weight, height, mean BMI, ASA score, tumor location from the anal verge, and operation history. None of these parameters differed significantly between the groups (Table 1).

**Table 1:** Patient characteristics (*n* = 180)

	Laparoscopic anterior resection (*n* = 146)	Robotic anterior resection (*n* = 34)	*p* value
Mean age: years (range)	59.7 ± 11.5 (29–90)	59.6 ± 8.4 (41–77)	0.969
Sex: *n* (%)			0.439
Male	87 (59.6)	23 (67.6)	
Female	59 (40.4)	11 (32.4)	
Mean weight: kg (range)	63.3 ± 11.9 (37–113)	66.7 ± 10.0 (48–93)	0.123
Mean height: cm (range)	163 ± 8 (135–184)	164 ± 9 (146–185)	0.481
Mean BMI: kg/m^2^ (range)	23.8 ± 3.8 (16.0–39.1)	24.8 ± 2.1 (20.8–29.4)	0.135
ASA score: *n* (%)			0.134
1	107 (73.3)	19 (55.9)	
2	33 (22.6)	13 (38.2)	
3	6 (4.1)	2 (1.4)	0.931
Mean tumor location from anal verge: cm (range)	25.9 ± 7.1 (13–50)	22.2 ± 7.2 (10–40)	
Previous operation history: *n* (%)	25 (17.1)	4 (11.7)	0.606

*BMI* body mass index, *ASA* American Society of Anesthesiologists

### Perioperative clinical outcomes and complications

The mean operating time was 217.6 ± 70.7 min (95 % confidence interval [CI], 205.6–229.1 min) in the L-AR group and 252.5 ± 94.9 min (95 % CI, 219.3–285.6 min) in the R-AR group (*p* = 0.016). The hemoglobin change did not differ significantly between the two groups (*p* = 0.546). Days to first gas passing and stool passing, days to soft diet, and days of hospitalization were statistically fewer in the R-AR group than in the L-AR group.

The L-AR group had one case of conversion compared with no conversions in the R-AR group. The reason for the conversion in the L-AR group was intraoperative bleeding due to an injury in the left renal vein.

The overall postoperative complication rate during fewer than 30 postoperative days was 10.3 % in the L-AR group and 5.9 % in the R-AR group (*p* = 0.281). The rate for severe complications requiring reoperation was 1.4 % in the L-AR group and 0 % in the R-AR group.

Readmission occurred for one patient each in the L-AR and the R-AR groups. The reason for readmission was ileus in both groups. There were no mortality cases in either group (Table 2).

**Table 2:** Perioperative clinical outcomes and postoperative complications

	Laparoscopic anterior resection (*n* = 146)	Robotic anterior resection (*n* = 34)	*p* value
Perioperative clinical outcomes			
Mean total operation time: min (range)	217.6 ± 70.7 (82–400)	252.5 ± 94.9 (117–460)	0.016
Mean intraoperative bleeding: ml (range)	78.2 ± 12.3 (0–600)	60.3 ± 27.0 (0–800)	0.772
Mean time to 1st gas passing: days (range)	2.52 ± 0.8 (1–5)	2.21 ± 0.9 (1–5)	0.040
Mean time to 1st stool passing: days (range)	4.42 ± 0.9 (2–7)	3.85 ± 0.2 (2–7)	0.003
Mean time to soft diet:days (range)	5.2 ± 1.3 (3–16)	4.5 ± 1.2 (2–7)	0.009
Mean hospital stay: (range)	6.2 ± 1.3 (4–17)	5.5 ± 1.6 (3–8)	0.005
Conversion			
Postoperative complications: *n* (%)			
Mild complications			
Chyloperitoneum	4 (2.7)	0 (0)	
Ileus	1 (0.7)	2 (5.9)	
Wound infection	2 (1.4)	0 (0)	
Voiding difficulty	5 (3.4)	0 (0)	
Moderate complications			
Pneumonia	0 (0)	0 (0)	
Postoperative bleeding	1 (0.7)	0 (0)	
Severe complications			
Intraabdominal abscess	0 (0)	0 (0)	
Anastomotic leakage	2 (1.4)	0 (0)	
Deaths (30-day mortality)	0 (0)	0 (0)	
Total no. of complications	15 (10.3)	2 (5.9)	0.281
Total no. of patients with complications	15 (10.3)	2 (5.9)	0.281
Readmission due to complications	1 (0.7)	1 (2.9)	0.258

### Postoperative pathologic results

The distribution of the TNM stage and the histologic grade of differentiation did not differ significantly between the two groups. The mean number of harvested lymph nodes was 16.5 ± 11.3 in the L-AR group and 12.0 ± 7.9 in the R-AR group (*p* = 0.031). The mean proximal resection margin was 8.2 ± 2.9 cm in the L-AR group and 10.7 ± 3.4 cm in the R-AR group (*p* = 0.026). The mean distal resection margin and the mass size did not differ significantly between the two groups (Table 3).

**Table 3:** Postoperative pathologic outcomes

	Laparoscopic anterior resection (*n* = 146)	Robotic anterior resection (*n* = 34)	*p* value
TNM stage: *n* (%)			0.185
1	52 (35.6)	14 (41.2)	
2	36 (24.7)	12 (35.3)	
3	58 (39.7)	8 (23.5)	
T stage: *n* (%)			0.954
1	38 (26.0)	9 (26.5)	
2	24 (16.4)	5 (14.7)	
3	78 (53.4)	19 (55.9)	
4	6 (4.1)	1 (2.9)	
N stage: *n* (%)			0.233
0	89 (61.0)	26 (76.5)	
1	48 (32.9)	7 (20.6)	
2	9 (6.1)	1 (2.9)	
Mean harvested lymph nodes: *n* (range)			
All stage	16.5 ± 11.3 (2–56)	12.0 ± 7.9 (1–31)	0.031
Stage 1	13.1 ± 11.9 (2–56)	7.7 ± 5.8 (1–21)	0.080
Stage 2	18.2 ± 8.8 (5–46)	15.5 ± 8.4 (3–26)	0.404
Stage 3	18.5 ± 11.5 (4–52)	14.4 ± 8.1 (5–46)	0.387
Mean PRM: cm (range)	8.2 ± 2.9 (3–19)	10.7 ± 3.4 (3.5–35)	0.026
Mean DRM: cm (range)	4.9 ± 2.8 (1–25)	5.4 ± 3.4 (1–20)	0.452

*TNM* tumor node metastasis, *PRM* proximal margin, *DRM* distal margin

### Oncologic outcomes

**Fig. 1:**
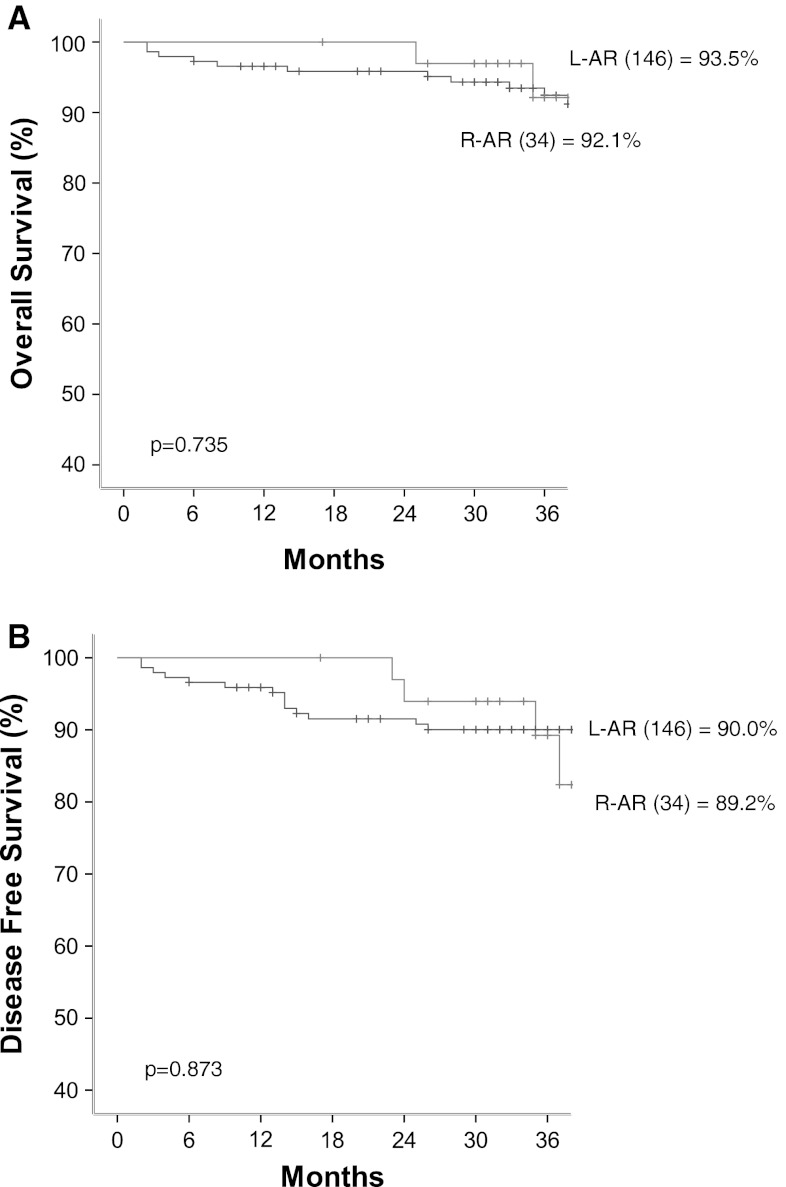
The 3-year overall (**A**) and 3-year disease-free (**B**) survival rates

**Fig. 2:**
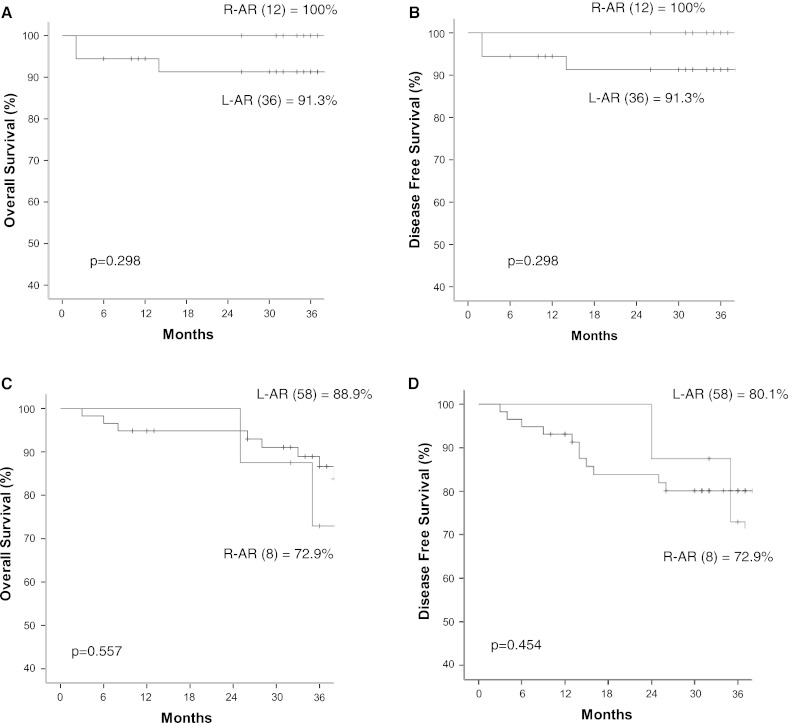
The 3-year overall and disease-free survival rates according to stage. **A** 3-year overall survival for stage 2. **B** 3-year disease-free survival for stage 2. **C** 3-year overall survival for stage 3 **D** 3-year disease-free survival for stage 3

The mean follow-up period was 36.9 ± 11.8 months (range, 2–63 months). The 3-year overall survival rate for the all the patients was 93.5 % in the L-AR group and 92.1 % in the R-AR group (*p* = 0.723). The 3-year disease-free survival rate for all the patients was 90.9 % in the L-AR group and 89.2 % in the R-AR group (*p* = 0.890) (Fig. 1). The 3-year overall and disease-free survival rate for the stage 1 patients was 100 % in both groups. The 3-year overall and disease-free survival rate for the stage 2 patients was 91.3 % in the L-AR group and 100 % in the R-AR group (*p* = 0.298) (Fig. 2A). The 3-year overall survival rate for the stage 3 patients was 88.9 % in the L-AR group and 72.9 % in the R-AR group (*p* = 0.557). The 3-year disease-free survival rate for the stage 3 patients was 80.1 % in the L-AR group and 72.9 % in the R-AR group (*p* = 0.454) (Fig. 2D).

## Discussion

The data from the study showed the feasibility and safety of R-AR, with oncologic and perioperative outcomes similar to those of L-AR. The mean operation time was significantly longer in the R-AR group than in the L-AR group. The short-term outcomes of this study showed patterns similar to those of previous published studies [8, 9, 13, 14].

During the postoperative course, the amount of intraoperative bleeding did not differ significantly between the R-AR and L-AR groups. However, the clinical parameters related to a fast recovery. The days to first gas passing, the days to first stool passing, the days to soft diet, and the days of hospitalization were significantly fewer in the R-AR group than in the L-AR group. These results showed a pattern similar to that in our previous report [6].

Core advanced technologies of the robotic system may have had a positive influence in the R-AR group. Relatively lower complication rates in the R-AR group than in the L-AR group also may be positively related to the technological advantages of the robotic system, although the difference was not statistically significant. Consequently, the relatively lower complication rates in the R-AR group may shorten the hospital stay, the time to first gas passing, and the time to first stool passing. However, the exact cause for the shorter hospital stay and lower complication rate cannot be assessed based on the study design.

Rawlings et al. [7] reported systematically on the advantages of robotic surgery including a view magnified tenfold, the surgeon’s control over the camera, seven degrees of freedom of the instrument tips, and reduced fatigue of the surgeon. These advantages may help to obtain better short-term clinical or long term oncologic outcomes than laparoscopically assisted surgery. However, a view magnified tenfold may not be necessary in the large surgical field for performing an anterior resection for sigmoid colon cancer. The surgeon’s control over the camera with a stable camera platform in robotic surgery can be compensated with an experienced camera holder in laparoscopic surgery. Two additional degrees of freedom of robotic instrument tips also can be compensated by proper traction and retraction of the redundant colon during mobilization of the colon.

The aforementioned considerations imply that a minimally invasive surgery expert can overcome the technological advantages of the robotic system by just using conventional laparoscopic instruments. The current generation of the robotic system has no voluntary action or decision and mimics the surgeon’s hand motion. This fact is the main reason for the difficulty evaluating the objective efficacy of the robotic system.

The 3-year overall survival and the 3-year disease-free survival of the R-AR group did not differ with those of the L-AR group. The oncologic outcomes according to the stages of R-AR showed results similar to those of the R-AR group.

The oncologic safety of laparoscopic colon surgery for cancer has been proven by large multicenter randomized clinical trials [1–3]. In these clinical trials, the oncologic results were comparable with those of open surgery. This implies that the resection range of laparoscopic colon surgery for cancer did not differ from that of open procedure in terms of resection margins, lymph node dissection, or any iatrogenic tissue injury of the resected specimen.

In the same manner, the quality of the resected specimen did not differ between the R-AR and L-AR groups in this study. The concepts of standard oncologic resection depend on the range of resection [15]. Thus, it cannot differ among open, laparoscopic, and robotic procedures. However, in this study, the total number of harvested lymph nodes was lower in the R-AR group and the proximal resection margin longer in the R-AR group than in the L-AR group.

The total number of harvested lymph nodes is related to understaging and poor oncologic outcomes [16]. In this study, the relatively small number of harvested lymph nodes in the R-AR group was not translated as poor oncologic outcomes compared with the L-AR group. The difference in the number of harvested lymph nodes may have been related to a type 2 statistical error considering the small number of cases in the R-AR group and the same resection range of both the robotic and the laparoscopic procedures. Further large-scale comparative studies are necessary to solve this issue.

The robotic system has several disadvantages despite its many advanced technological advantages. The robotic system has no tactile sense. Thus, all instruments should be moved very precisely and carefully in the small surgical field generated by the camera to prevent dangerous injuries around the site. Moreover, the operator cannot know the power of holding the tissue and the tension of traction or countertraction during the procedure. Just a visual cue can compensate for this disadvantage.

These disadvantages can be related to the longer operation time in the R-AR group compared with that in the L-AR group in this study because fast instrument movement and movement around the site are possible with the laparoscopic procedure, which has tactile sense. Docking time of robotic instruments and disengagement can be another explanation for the longer operation time in the R-AR group. Similarly, with this study, Rawlings et al. [17] reported a longer operation time for the robotic procedure than for the laparoscopic procedure although the data did not reach statistical significance. However, the operation time difference of 35 min on the average (~14 % of the operation length) can be considered nonsignificant for a 4-h operation in this study.

## Conclusion

Compared with L-AR, R-AR was technically safe and feasible and had similar oncologic safety and perioperative outcomes. No extra-significant morbidity or mortality was noted in the R-AR group. However, the high cost might be a further debatable issue for general adaption of R-AR.

This study had a potential selection bias because randomization was not performed and the study had a retrospective case–control design. Thus, future large-scale randomized clinical trials and objective cost-effectiveness analysis should evaluate the efficacy of robotically assisted colon surgery.
